# Effect of Silver Nanoparticle Size on Antibacterial Activity

**DOI:** 10.3390/toxics12110801

**Published:** 2024-11-05

**Authors:** Vadim A. Ershov, Boris G. Ershov

**Affiliations:** Frumkin Institute of Physical Chemistry and Electrochemistry, Russian Academy of Science, 119071 Moscow, Russia; ershov@ipc.rssi.ru

**Keywords:** silver nanoparticles, antibacterial material, toxicity, *Escherichia coli*, *Staphylococcus aureus*, hazard

## Abstract

The ubiquitous use of products containing AgNPs results in the entry of nanoparticles into the environment. Both nanoparticles and Ag^+^ released upon their oxidative dissolution have a toxic effect on living microorganisms. The antibacterial activity of spherical silver nanoparticles of 10.8 ± 0.8 nm and 22.7 ± 2.2 nm in size stabilized by carbonate ions was studied against *Escherichia coli* and other bacteria. The biocidal action of silver increases as the particle size decreases. Analysis of these results and other known data made it possible to substantiate a linear proportional relationship between the minimum inhibitory concentration (MIC) or the half-maximal inhibitory concentration (IC50) and silver nanoparticle size and determine empirical parameters for this relationship. The antibacterial activity (toxicity) is directly proportional to the specific surface area of nanosized silver.

## 1. Introduction

The extensive use of silver is due to its high biocidal activity and ability to suppress harmful microflora. This behavior was detected and widely used in ancient times to extend the safe storage time for water and food. Currently, it has been shown that silver nanoparticles have a clear-cut action against bacteria, viruses, and biofilms and possess a fungicidal activity [1,2,3,4]. This accounts for the use of silver in various fields of technology, medicine, agriculture, and the food industry. However, this brings about a serious problem related to the fact that the migration and transformation of nanoparticles in the environment have toxic effects on natural microbial communities and habitats. Entry into the aquatic environment, which is the most likely destination upon scattering, is harmful to living organisms and poses a real environmental hazard [5,6,7]. Elucidation of the regularities of nanoparticle influence on the microflora is important for risk evaluation and prediction of various environmental hazards [8,9]. It was shown [2,10,11,12,13] that the particle size influences the antibacterial activity of silver. As the size decreases, the toxicity markedly increases.

The goal of this study is to analyze the size effect of the antibacterial activity of AgNPs and elucidate and substantiate the quantitative size–activity relationship. A solution to this important problem would make it possible both to predict the antibacterial activity of silver nanoparticles against various representatives of microflora and substantiate the strategy for considering their toxic action in the environment. In addition, the obtained results may be useful for predicting the strength of antibacterial effects that may occur when using medical materials containing silver.

## 2. Results and Discussion

**Figure 1:**
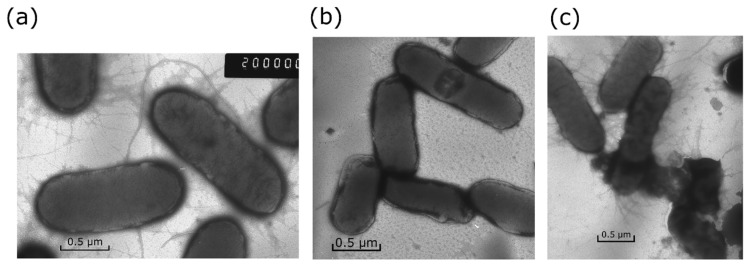
Micrographs of *Escherichia coli*: (**a**) control; (**b**) grown in the presence of 1 × 10^−5^ mol L^−1^ of Ag^+^ ions; (**c**) grown in the presence of carbo-AgNPs (D (2R) = 22.7 nm, 1 × 10^−5^ mol L^−1^ of Ag^0^).

In our studies, we used silver nanoparticles stabilized by carbonate ions (carbo-AgNPs). The preparation method and characteristics of these particles were described in detail earlier, and an investigation of their biocidal action on *Escherichia coli* and other bacteria was carried out by the procedure described in [14]. Spherical carbo-AgNPs with sizes of 10.8 ± 0.8 nm and 22.7 ± 2.2 nm were used. The ICP-MS method, Element 2 (Thermo-Finnigan, Bremen, Germany), was used to determine the concentration of dissolved Ag^+^ in the nutrient medium. To separate ions from nanoparticles, the nutrient medium with silver was centrifuged at 14,600 rpm for 30 min at Eppendorf Centrifuge 5424 (Eppendorf AG, Hamburg, Germany). After that, the optical spectra of the samples were measured. It was found that the bands of the optical spectrum characteristic of silver nanoparticles were absent, which indicates the removal of nanoparticles and the possibility of further determination of silver exclusively in ionic form. An important advantage of the study of their influence on the bacteria is that they contained no polymer stabilizers, which are often used, and their role in the biocidal action is always difficult to take into account. Electrostatic stabilization was performed by carbonate ions, which are typical and safe components of the natural aquatic environment. Carbo-AgNPs have high activity against *Escherichia coli*, gram-negative *Pseudomonas putida*, and gram-positive *Paenibacillus jamilae* bacterial cells. Figure 1 shows a comparison of the effects of Ag^+^ ions and carbo-AgNPs (for the same concentration of Ag^+^ ions and, correspondingly, Ag^0^ atoms equal to 1 × 10^−5^ mol L^−1^ at the same experimental time of 48 h) on the *Escherichia coli* bacterial cells. The *Escherichia coli* cells grown without silver have a smooth oval shape and have flagella and numerous pili, which are responsible for the transfer of genetic material and adhesion (Figure 1a). In the presence of Ag^+^ ions, the main morphological sign of their effect is an almost complete loss of pili and flagella (Figure 1b). In general, the *Escherichia coli* cells do not significantly change in shape and retain membrane integrity. The presence of carbo-AgNPs of 10.8 nm and 22.7 nm in size markedly changes the cell morphology (Figure 1c). A partial loss of pili and a pronounced destruction of cell membranes take place. Cell damage (destruction) is the most obvious sign of the effect of silver nanoparticles.

The ICP-MS method was used to determine the concentration of Ag^+^ ions released into the solution as a result of the oxidation of silver nanoparticles. It was found that about 25% of the total amount of metal (2.5 × 10^−^^6^ mol L^−^^1^) passed into solution as Ag^+^ ions during 48 h of the experiment. The data in Figure 1b show that even a high concentration of Ag^+^ ions causes a loss of pili but does not lead to cell destruction. Therefore, the comparison of Figure 1b,c allow us to draw an unambiguous conclusion that the pronounced destruction of cells is caused, first of all, by silver nanoparticles adsorbed on them.

These results attest to the presence of a specific mechanism of toxicity of silver nanoparticles in comparison with Ag^+^ toxicity. Apart from the inhibition of development of *Escherichia coli* cells inherent in Ag^+^ ions (indirect action), nanoparticles are characterized by a specific contact mechanism of cell damage, which leads to cell destruction (direct action) [14]. However, it is necessary to bear in mind that the inhibition of bacteria by both Ag^+^ ions and Ag nanoparticles occurs only in aerated solutions, that is, in the presence of air oxygen. Particularly, under these conditions, the oxidative dissolution of nanoparticles with the release of Ag^+^ ions takes place. This strongly suggests that in a local contact area, the oxidative dissolution of silver and the formation of reactive oxygen species ($O_{2}^{-•}$/${HO}_{2}^{•}$ and ^•^OH radicals and H_2_O_2_) result in the chemical degradation of the protective cell membrane of *Escherichia coli* and the destruction of the cell [14]. The degree of oxidative dissolution of nanoparticles noticeably increases with decreasing nanoparticle size [15]. Therefore, one may expect a similar size effect on biocidal activity.

**Figure 2:**
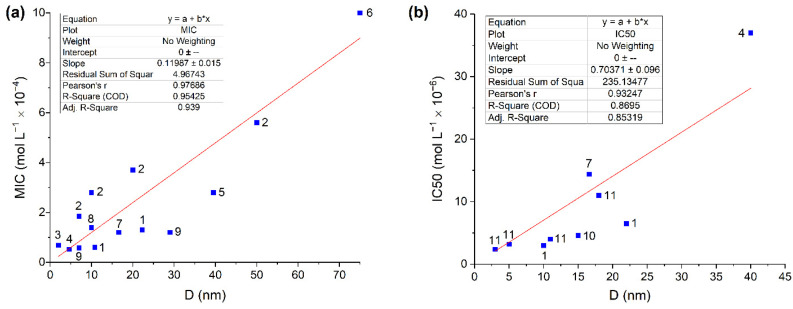
Dependences of (**a**) MIC and (**b**) IC50 for the inhibition of *Escherichia coli* cell growth on AgNP size. The presented values (■) were taken from the following publications: 1—[14], 2—[11], 3—[16], 4—[17], 5—[18], 6—[19], 7—[20], 8—[21], 9—[12], 10—[22], 11—[13]. Tables of data and conditions are given in Tables S1 and S2.

It was shown that the specific biocidal effect referred to as the concentration of silver atoms in the form of carbo-AgNPs markedly increases with decreasing particle size. This was noted in the determination of the minimum inhibitory concentration (MIC), i.e., the lowest concentration of an antimicrobial ingredient or agent that is bacteriostatic (prevents bacterial growth). The same trend was revealed for the half-maximal inhibitory concentration (IC50), that is, the concentration required to inhibit the development of microflora by 50%. MIC and IC50 were determined by mathematical calculations in the OriginPro program (non-linear growth/sigmoidal curve fitting) [14]. It was shown that for particles of 22.7 nm in size, MIC and IC50 are approximately 1.3 × 10^−4^ mol L^−1^ and 6.5 × 10^−6^ mol L^−1^, while for 10.8 nm particles, these values decrease to 0.6 × 10^−4^ mol L^−1^ and 3.0 × 10^−6^ mol L^−1^, respectively. This correlates well with the results of a study of the size effect [11], in which MIC was found to regularly increase in the order 1.85 × 10^−4^ mol L^−1^, 2.8 × 10^−4^ mol L^−1^, 3.7 × 10^−4^ mol L^−1^, and 5.6 × 10^−4^ mol L^−1^ as the silver nanoparticle size increased in the order of 7 nm, 10 nm, 20 nm, and 50 nm. A similar trend was observed for the variation of IC50 with the increasing size of silver nanoparticles. For 3 nm, 5 nm, 11 nm, and 18 nm particles, IC50 successively increased by 2.4 × 10^−6^ mol L^−1^, 3.2 × 10^−6^ mol L^−1^, 4.0 × 10^−6^ mol L^−1^, and 11.0 × 10^−4^ mol L^−1^, respectively [13]. Unfortunately, there are only few systematic data of this type in the literature; researchers mainly restrict themselves to the analysis of the biocidal activities of silver nanoparticles of one or two sizes against *Escherichia coli* cells. However, the whole set of experimental data shows quite evident and reliable linear proportional dependences of MIC and IC50 on the nanoparticle size (Figure 2). Pearson’s correlation coefficient is 0.98 (for MIC) and 0.93 (for IC50).

Note that MIC and IC50 values characterize the antibacterial activity of silver, i.e., its ability to inhibit cell viability. The lower the MIC and IC50, the higher the antibacterial activity of AgNPs. It can be seen in Figure 2 that despite the differences in the AgNP prehistory, size distribution, or distributions of other properties, the dependence of the bacterial activity of AgNPs on the particle size can be clearly seen and is described by simple empirical equations as follows:

[MIC] = *k_mic_* × D (1)

[IC50] = *k_IC50_* × D, (2)

where *k_mic_* = 1.4 × 10^−5^ mol L^−1^ nm^−1^ and *k_IC50_* = 9.1 × 10^−7^ mol L^−1^ nm^−1^.

**Figure 3:**
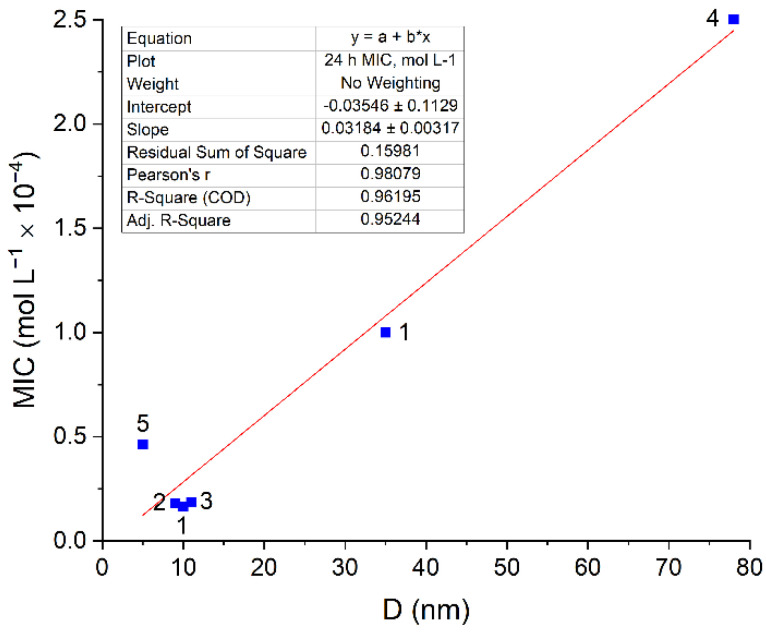
Dependence of MIC for the inhibition of *Staphylococcus aureus* cell growth on the size of AgNPs. The presented values (■) were taken from the following publications: 1—[23], 2—[24], 3—[25], 4—[26], 5—[27]. Tables of data and conditions are given in Table S3.

Apparently, nanosilver exerts a similar antibacterial effect on other types of microflorae. In this study, AgNPs were found to inhibit gram-negative *Pseudomonas putida* and gram-positive *Paenibacillus jamilae* bacterial cells. As the nanoparticle size increased from 10.8 nm to 23.1 nm, MIC for *Pseudomonas putida* increased from 5 × 10^−5^ mol L^−1^ to 1 × 10^−4^ mol L^−1^, while IC50 for *Paenibacillus jamilae* increased from 3.3 × 10^−7^ mol L^−1^ to 1.1 × 10^−6^ mol L^−1^. Regarding *Staphylococcus aureus*, there is a possibility to elucidate the relationship between the biocidal action on the microflora and the particle size for AgNPs in more detail by comparing the results of various studies [23,24,25,26]. The results of the analysis show the same linear proportionality of MIC on the nanoparticle size (Figure 3) for *Escherichia coli* (Figure 2). Pearson’s correlation coefficient is 0.98.

The resulting *k_mic_* is 3.2 × 10^−6^ mol L^−1^ nm^−1^. A comparison with the data for *Escherichia coli* (*k_mic_* = 1.4 × 10^−5^ mol L^−1^ nm^−1^) indicates that *Staphylococcus aureus* is more stable against the toxic action of silver. This result confirms a previously identified pattern: gram-positive bacteria, such as *Staphylococcus aureus,* are more resistant to AgNPs due to their thick cell wall composed of a peptidoglycan layer compared to gram-negative bacteria, such as *Escherichia coli* [28,29].

The set of presented data definitely indicates the presence of a reliable linear dependence of the concentrations of silver atoms in nanoparticles providing a detectable biocidal effect (MIC or IC50) against various bacterial species on the particle size. As the size grows, the concentration providing damage to bacteria increases, i.e., the antibacterial effect of silver decreases. Conversely, the smaller the nanoparticle size, the more pronounced the biocidal effect since the specific surface area is greater for smaller particles. We will analyze the established regularity and elucidate the meaning behind the relationship between MIC or IC50 and R. The linear-proportional dependence of MIC on R implies the invariability of *k_mic_* = MIC/R. The expected biocidal effect of bacterial damage for nanoparticles of different sizes occurs provided that the MIC/R ratio is equal to *k_mic_*. Recall that MIC and IC50 are not concentrations of silver atoms homogeneously distributed throughout the solution. In fact, these atoms make up silver particles of various sizes. We will express MIC in terms of the total volume of the silver nanoparticles as *MIC_v_*. Then, the concentration of nanoparticles (*n*) with the radius *R* is given by

(3) $$n=\frac{{MIC}_{v}}{\left[\left(\frac{4}{3}\right)\pi R^{3}\right]}$$

From this, it follows that

(4) $$k_{mic}=\frac{{MIC}_{v}}{R}=\frac{n\cdot [\left(\frac{4}{3}\right)\pi R^{3}]}{R}=\left(\frac{1}{3}\right)\cdot n\times 4\pi R^{2},$$

that is, it is equal to the product of the number of silver particles n and the surface area of the sphere 4π*R*^2^. Hence, the linear proportional dependence of *MIC* on *R* has a clear and definite meaning. This is the proportionality between the total area of nanoparticles in the bulk solution and the particle size. The biocidal effect for nanoparticles of different sizes is attained when their surface area is equal to or greater than the critical value as follows:

(5) $$\left(\frac{1}{3}\right)\times n\times 4\pi R^{2},$$

i.e., the value of *k_mic_*. This means that when particles of different sizes attain a toxic antibacterial effect, they have the same total surface area. This is quite understandable and, to some extent, an obvious meaning. Indeed, the toxic effect of silver inhibiting the viability of microflora is, most likely, attributable to the Ag^+^ ions released upon the oxidative dissolution of silver particles [15,30,31,32]. Silver nanoparticles are dissolved by an electrochemical mechanism, with the degree of dissolution being dependent on the particle size [15]. The surface area of nanoparticles naturally determines the concentrations of silver ions released into the solution. The Ag^+^ ions released to the bulk solution upon the oxidative dissolution of nanoparticles get involved in biochemical processes, thus inhibiting the viability of cellular structures (indirect action). The direct contact of nanoparticles with a bacterium generates increased local concentrations of Ag^+^ ions and reactive oxygen species (ROS), which cause oxidative stress (direct action). The effect of direct action is clearly seen in the examination of cell morphology as a process leading to cell death (Figure 1).

It can be concluded that the antibacterial activity (toxicity) is directly proportional to the specific surface area of nanosized silver. The smaller the particle size, the greater the specific surface area of the dispersion and the higher the efficiency of silver in inhibiting the bacterial microflora. The increase in the biocidal effect of silver particles with a decrease in their size can be caused by several reasons. The fraction of dissolved metal and the rate of release of Ag^+^ ions into the solution increase with a decrease in AgNP size [ccылки расtboреhиe]. Formed Ag^+^ inhibits respiration by interacting with the thiol (-SH) group of cysteine, forming an S-Ag bond [33]. Additionally, the smaller the particle size, the more effective it is at penetrating bacterial cells [34]. Another characteristic of the toxicity of silver nanoparticles is their ability to cause oxidative stress. This mechanism occurs mainly through direct contact with bacteria and high intracellular concentrations of ROS. Smaller particles have a larger specific surface area, which increases their contact area with bacteria.

## Data Availability

The manuscript contains a detailed presentation of the study’s novel findings. Supplementary Materials offer further supporting data. For more information, please contact the corresponding author.

## Supplementary Materials

The following supporting information can be downloaded at https://www.mdpi.com/article/10.3390/toxics12110801/s1, Table S1: MIC of AgNPs of different sizes in relation to *Escherichia coli*; Table S2: IC50 of AgNPs of different sizes in relation to *Escherichia coli*; Table S3: MIC of AgNPs of different sizes in relation to *Staphylococcus aureus*.
