# Supplementary material for: Effect of Silver Nanoparticle Size on Antibacterial Activity
Source: Toxics. 2024 Nov 5;12(11):801. doi: 10.3390/toxics12110801 (PMC11598259; doi:10.3390/toxics12110801)
Supplement: Supplementary file 1 [file toxics-12-00801-s001.zip › toxics-3220147-supplementary.pdf]

Supplementary materials

Effect of Silver Nanoparticle Size on the Antibacterial Activity

Vadim Ershov* and Boris Ershov

Frumkin Institute of Physical Chemistry and Electrochemistry, Russian Academy of Science, Leninsky Pr. 31-4,119071 Moscow, Russia; ershov@ipc.rssi.ru
* Correspondence: vadersh@yandex.ru

Table S1. MIC of AgNPs of different sizes in relation to *Escherichia coli*.

Number on figure	D (nm)	Surface functionalization	MIC (mol $L^{-1} \times 10^{-4}$)	Reference (same as in the manuscript)
1	10.8<br>22.3	Carbonate	0.6<br>1.3	[14]
2	7<br>10<br>20<br>50	Citrate	1.85<br>2.8<br>3.7<br>5.6	[11]
3	2	Biosynthesized by *Fusarium scirpi*	0.69	[16]
4	4.65	Citrate	0.52	[17]
5	39.5	PVP, M_W = 55000	2.8	[18]
6	75	PVP, M_W = 40000	10	[19]
7	16.6	Biostabilization	1.2	[20]
8	10	Citrate	1.4	[21]
9	7<br>29	Gallic acid	0.58<br>1.2	[12]

Table S2. IC50 of AgNPs of different sizes in relation to *Escherichia coli*.

Number on figure	D (nm)	Surface functionalization	IC50 (mol $L^{-1} \times 10^{-6}$)	Reference (same as in the manuscript)
1	10.8<br>22.3	Carbonate	3<br>6.5	[14]
4	40	Citrate	37	[17]
7	16.6	Biostabilization	14.4	[20]
8	10	Citrate	46	[21]
10	15	PVA	4.6	[22]
11	3<br>5<br>11	Glycol-thiol	2.4<br>3.2<br>4<br>11	[13]

Table S3. MIC of AgNPs of different sizes in relation to *Staphylococcus aureus*.

Number on figure	D (nm)	Surface functionalization	MIC (mol $L^{-1} \times 10^{-4}$)	Reference (same as in the manuscript)
1	10<br>35	n/a	0.17<br>1.0	[23]
2	9	PVA	0.18	[24]
3	11	Quercetin	0.19	[25]
4	78	Biosynthesized by *F. oxysporum*	2.5	[26]
5	5	n/a	0.46	[27]
